# Translational switching of Cry1 protein expression confers reversible control of circadian behavior in arrhythmic Cry-deficient mice

**DOI:** 10.1073/pnas.1811438115

**Published:** 2018-11-28

**Authors:** Elizabeth S. Maywood, Thomas S. Elliott, Andrew P. Patton, Toke P. Krogager, Johanna E. Chesham, Russell J. Ernst, Václav Beránek, Marco Brancaccio, Jason W. Chin, Michael H. Hastings

**Affiliations:** ^a^Division of Neurobiology, Medical Research Council Laboratory of Molecular Biology, CB2 0QH Cambridge, United Kingdom;; ^b^Division of Protein and Nucleic Acid Chemistry, Medical Research Council Laboratory of Molecular Biology, CB2 0QH Cambridge, United Kingdom

**Keywords:** circadian, genetic code expansion, cryptochrome, amber suppression, suprachiasmatic nucleus

## Abstract

The suprachiasmatic nucleus (SCN) is the principal circadian clock of mammals, coordinating daily rhythms of physiology and behavior. Circadian timing pivots around self-sustaining transcriptional–translational negative feedback loops (TTFLs), whereby CLOCK and BMAL1 drive the expression of the negative regulators Period and Cryptochrome (Cry). Global deletion of Cry1 and Cry2 disables the TTFL, resulting in arrhythmicity in downstream behaviors. We used this highly tractable biology to further develop genetic code expansion (GCE) as a translational switch to achieve reversible control of a biologically relevant protein, Cry1, in the SCN. This employed an orthogonal aminoacyl-tRNA synthetase/tRNA_CUA_ pair delivered to the SCN by adeno-associated virus (AAV) vectors, allowing incorporation of a noncanonical amino acid (ncAA) into AAV-encoded Cry1 protein carrying an ectopic amber stop codon. Thus, translational readthrough and Cry1 expression were conditional on the supply of ncAA via culture medium or drinking water and were restricted to neurons by synapsin-dependent expression of aminoacyl tRNA-synthetase. Activation of Cry1 translation by ncAA in neurons of arrhythmic Cry-null SCN slices immediately and dose-dependently initiated TTFL circadian rhythms, which dissipated rapidly after ncAA withdrawal. Moreover, genetic activation of the TTFL in SCN neurons rapidly and reversibly initiated circadian behavior in otherwise arrhythmic Cry-null mice, with rhythm amplitude being determined by the number of transduced SCN neurons. Thus, Cry1 does not specify the development of circadian circuitry and competence but is essential for its labile and rapidly reversible activation. This demonstrates reversible control of mammalian behavior using GCE-based translational switching, a method of potentially broad neurobiological interest.

## Significance

Circadian rhythms dominate our lives through our daily cycle of sleep and wakefulness. They are controlled by a brain master clock: the suprachiasmatic nucleus (SCN). SCN timekeeping pivots around a molecular loop incorporating Cryptochrome (Cry) proteins; global loss of these proteins disables the clock. We developed a biologically appropriate translational switch based on genetic code expansion to achieve reversible control of Cry1 expression. Cry1 translation in neurons of arrhythmic Cry-null SCN slices immediately, reversibly, and dose-dependently initiated circadian molecular rhythms. Cry1 translation in SCN neurons was sufficient to initiate circadian behavior rapidly and reversibly in arrhythmic Cry-null mice. This demonstrates control of mammalian behavior using translational switching, a method of broad applicability.

Circadian rhythms in mammalian cells are driven by cell-autonomous, self-sustaining transcriptional–translational negative feedback loops (TTFLs) whereby CLOCK and BMAL1 drive the expression of the negative regulators Period (Per) and Cryptochrome (Cry) (1). The suprachiasmatic nucleus (SCN) of the hypothalamus is the principal circadian pacemaker (2). It directs the innumerable cell-autonomous TTFL-based oscillations across the organism by a variety of SCN-dependent systemic cues derived from daily autonomic, behavioral, and endocrine rhythms (3). Circuit-level synchronization within the SCN is achieved via interneuronal, neuropeptidergic interactions as well as by neuronal–astrocytic communication, which act together to enhance the amplitude of the TTFL and so confer robustness to the SCN network and its dependent rhythms (4). Global deletion of Cry1 and Cry2 disables the TTFL in cells and tissues and abrogates circadian physiological and behavioral rhythms at the level of the animal (5), while mutations in Cry proteins have been associated with human sleep disorders (6, 7).

To better understand the role of Cry proteins in controlling circadian behavior, we sought to develop a method that would confer conditional, rapid, and reversible control of Cry expression in SCN cells of otherwise “clockless” Cry-deficient mice. The development of new tools to facilitate the conditional manipulation of gene expression has significantly expanded the understanding of neuronal cell biology and the genetic specification of behavior (8); the most commonly used methods target genes of interest via intersectional approaches involving genomic and virally mediated [commonly, adeno-associated virus (AAV)] manipulations. However, the effects of such Cre-loxP and Flp-FRT systems are permanent and may have unanticipated off-target consequences that require carefully controlled experimental design (9). More sophisticated, temporally specific control of transcription has been achieved using versions of the ligand-binding domain of the estrogen receptor (ER) to drive recombinase activity following the delivery of synthetic steroid, while truly reversible inducible approaches have been developed using the tetracycline (tet)-operon/repressor bitransgenic system (10, 11). Indeed, various genomic transcriptional approaches have been applied successfully to interrogate the TTFL circadian functions of Bmal1 (12), Cry1 (13), and Per2 (14).

To complement and extend this transcriptional toolkit, we sought to build on our recent development of a “translational switch” based on genetic code expansion (GCE). Translational switching employs an orthogonal aminoacyl-tRNA synthetase/tRNA_CUA_ pair to incorporate a noncanonical amino acid (ncAA) into a protein of interest at an ectopic amber stop codon (15). This allows translational readthrough and expression of the full-length protein of interest that is conditional on the supply of ncAA. As proof of principle, we have recently applied variants of this approach to express EGFP (16) in the mouse brain and to label and identify cell-type–specific brain proteomes (17). Here, we developed translational switching to achieve ncAA-dependent expression of Cry1, a biologically relevant protein, in SCN neurons. We show conditional, reversible control of molecular and behavioral circadian rhythms in Cry-deficient mice using GCE-mediated translational switching. Importantly, the initiated rhythms had circadian periods definitive of a Cry1-competent, Cry2-null TTFL, providing explicit proof of their control by translationally regulated Cry1. This GCE-based approach provides both an alternative and a complement to methods that employ transcriptional switching to control the expression of genes of interest and may be of broad neurobiological utility. Moreover, it shows that reinstating clock function in the SCN alone is sufficient to initiate and sustain circadian behavior in a clock-incompetent mouse and that this effect can be mediated solely by neurons in the SCN, with a period that can be tuned dose-dependently by the supply of ncAA and consequent level of Cry1 expression.

## Results

**Fig. 1.**
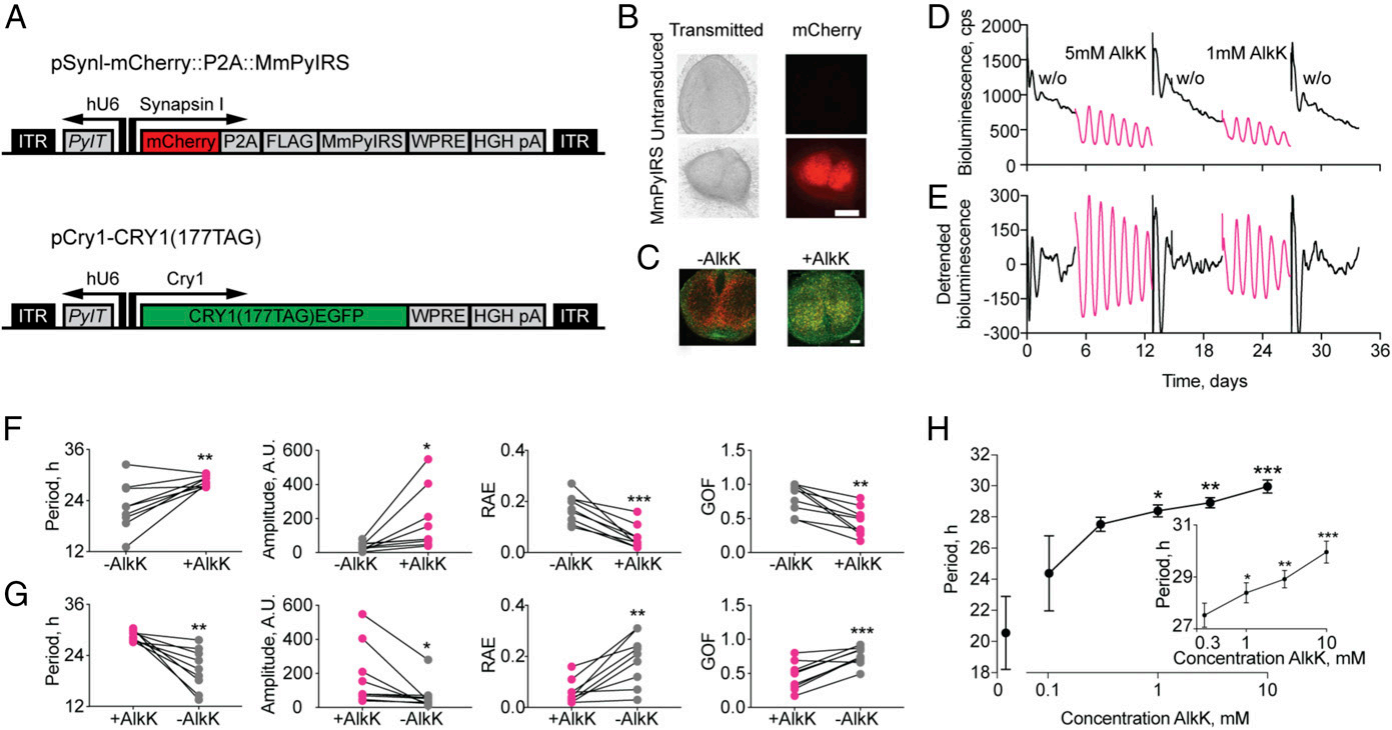
Translational switching of Cry1 expression controls molecular circadian rhythms in organotypic Cry-null SCN slices. (*A*) Schematic views of the AAV constructs used to deliver the orthogonal aminoacyl tRNA synthetase/tRNA pair and the target Cry1(_177TAG_)::EGFP. (*B*) Phase (*Left*) and fluorescence (*Right*) views of control (*Upper*) and AAV-transduced (*Lower*) SCN slices to show activity of the mCherry reporter. (Scale bar: 250 μm.) (*C*) Representative photomicrographs of AAV-transduced SCN slices taken before (−AlkK) and 34 h after treatment with 1 mM AlkK (+AlkK). The yellow signal in the AlkK-treated slice reveals the colocalization of the mCherry and Cry1::EGFP signals. (Scale bar: 100 μm.) (*D*) Representative plot of Per2::Luc bioluminescence from a Cry1,2-null SCN slice cotransduced with AAVs as in *A* and then treated with two sequences of AlkK addition followed by washout (w/o) with fresh medium. (*E*) Detrended plot of data in *D*. (*F*) FFT-determined periods, amplitude, RAE, and GOF of bioluminescence rhythms of SCN slices before and during the addition of AlkK. *P < 0.05, **P < 0.01, ***P < 0.001, paired *t* test. (*G*) As in *F*, for SCN slices during and after withdrawal of AlkK. *P < 0.05, **P < 0.01 paired *t* tests. (*H*) Dose–response curve showing the increase in period with increasing concentration of AlkK. *P < 0.05, **P < 0.01, ***P < 0.001 vs. 0.1 mM AlkK, one-way ANOVA.

To test the potential of translational switching for conditional expression of Cry1, SCN organotypic slices were cotransduced with two AAV-expressed constructs (Fig. 1*A*). The first was a previously validated generic AAV containing the genes for the orthogonal pyrrolysyl-tRNA_CUA_ (*PylT*) under the hU6 promoter and the orthogonal pyrrolysyl-tRNA synthetase PylRS (*PylS*) alongside an mCherry expression reporter, both under the neuronally specific hSynapsin promoter (16). We developed a second vector, AAV pCry1-Cry1(_177TAG_)::EGFP, encoding our protein of interest, Cry1, with a C-terminal EGFP tag to visualize expression. For circadian control, Cry1 is expressed from the minimal *Cry1* promoter (18, 19), and for translational conditionality it carries a TAG mutation to confer amber dependence (a second copy of the *PylT* is also provided in this vector). These AAVs allow the incorporation of the ncAA alkyne lysine *N6*-[(2-propynyloxy)carbonyl]-l-lysine (AlkK), the substrate for PylRS (20), into Cry1. When present, this permits translational readthrough at the amber stop codon, leading to the expression of full-length Cry1::EGFP in an AlkK-dependent manner.

### Translational Switching of Cry1 Expression in SCN Slices in Vitro.

Nonconditional (amber-free) AAV-mediated expression of pCry1-Cry1::EGFP can induce circadian bioluminescence rhythms in arrhythmic Cry1,2-null SCN (19). To test the potency of pCry1-Cry1(_177TAG_)::EGFP delivered by translational switching, Per2::Luc bioluminescence was first recorded from neonatal [postnatal day (P)10] Cry1,2-null SCN to confirm the absence of circadian oscillations. The slices were then transduced with the two AAVs and were cultured for 7–10 d; mCherry expression was used to demonstrate activity of the AAV encoding the synthetase/tRNA pair (Fig. 1*B*). In the absence of AlkK there was no EGFP report of Cry1 expression (Fig. 1*C* and *SI Appendix*, Fig. S1*A*), consistent with a lack of translationally switched Cry1 expression, and the level of Per2-driven bioluminescence was high, reflecting the absence of Cry-mediated repression of CLOCK/BMAL1-mediated activation of Per2 (Fig. 1*D* and *SI Appendix*, Fig. S1 *A* and *B*). Moreover, the AAV-transduced SCN tissue did not exhibit circadian rhythmicity (Fig. 1 *D* and *E*), although fast Fourier transform (FFT) analysis within BioDare2 was able to assign nominal noncircadian periods to the traces. These ranged between 12 and 33 h (22.7 ± 1.9 h, mean ± SEM; *n* = 9) (Fig. 1*F*), were of very low amplitude, and had a high relative amplitude error (RAE) indicative of poor circadian coherence (Fig. 1*F*).

When AlkK (1–5 mM) was added to slices, Cry1 expression (reported by EGFP fluorescence) was induced (Fig. 1*C*), and there was an immediate decrease in the level of Per2-driven bioluminescence, which was observed consistently both within a slice on repeated treatments and between slices (Fig. 1*D* and *SI Appendix*, Fig. S1*B*). This rapid decline, evident over 6 h, was a qualitative indicator of the potent repressor effect of newly translated Cry1. To obtain a more quantitative assessment of this suppressive activity, the changes in Per2::Luc bioluminescence were normalized for comparison with slices treated with 1 mM AlkK, and the rate of decline was calculated to be 29.1 ± 1.6% (*n* = 7) over 3 h, that is, *ca*. 10%/h (*SI Appendix*, Fig. S1*C*, initiation). Following this initial decline, Per2::Luc bioluminescence started to increase and then oscillate with a clearly defined circadian pattern, the peak levels of Per2 being comparable to, but not exceeding, the levels before the addition of AlkK. Therefore the overall effect of translationally switched Cry1, which was rhythmically expressed (as revealed by confocal imaging of SCN slices taken at the peak vs. trough of Per2::Luc bioluminescence) (*SI Appendix*, Fig. S1*B*), was to introduce rhythmic negative regulation into the TTFL. The period of the newly initiated oscillations converged across slices at 28.5 ± 0.4 h (1 mM AlkK, *n* = 9). This was significantly longer than the nominal periods observed before the addition of AlkK, *t*(8) = 3.4, *P* < 0.01, paired *t* test within slices (Fig. 1*F*); being >24 h, it is characteristic of a Cry1-driven, Cry2-null SCN [as compared with periods of 26.2 ± 0.1 h in the genomic mutant (21) and 26.0 ± 0.2 h in nonconditional pCry1-Cry1::EGFP AAV-mediated rhythm (19)]. Furthermore, the amplitude, the RAE, and the goodness of fit (GOF) of the molecular oscillations were significantly improved in the presence of AlkK (Fig. 1*F*): amplitude, pretreatment = 35.6 ± 7.8 vs. +AlkK = 181.2 ± 59.9, *t*(8) = 2.6, *P* < 0.05; RAE, pretreatment = 0.17 ± 0.02 vs. +AlkK = 0.06 ± 0.02, *t*(8) = 6.1, *P* < 0.001; GOF, pretreatment = 0.8 ± 0.07 vs. +AlkK = 0.46 ± 0.07, *t*(8) = 4.9, *P* < 0.001; all *n* = 9, paired *t* tests. The coherent bioluminescence rhythms persisted as long as AlkK was present. When slices were treated with a range of AlkK concentrations, the period of the oscillation was dose-dependent (*n* = 5–10 slices per concentration; slices in Fig. 1 *F* and *G* were incorporated into the dose–response curve in Fig. 1*H*). AlkK at the lowest dose tested (0.1 mM) was minimally effective at initiating circadian cycles, but as the concentration increased between 0.3 and 10 mM, AlkK initiated well-defined oscillations, and their period also lengthened systematically and dose-dependently within a range of *ca*. 2.5 h (27.5–30 h) (Fig. 1*G*, *Inset*). This dose-dependent increase in period was paralleled by a dose-dependent increase in the concentration of Cry1 protein after the addition of 0, 0.3, or 3 mM AlkK (*SI Appendix*, Fig. S1*A*), as assessed by the background-subtracted ratio of EGFP (as a proxy of Cry1 concentration) to mCherry measured in 40 cells from each of two separate fields of view (confocal image at 63× magnification) from four to six independent SCN slices per concentration (*n* = 15). Thus, the incorporation of AlkK into Cry1 does not compromise its role as a defining component within the SCN circadian clockwork.

The initial suppression of Per2 caused by newly translated Cry1 was significantly more rapid than the spontaneous decline observed after the first peak of the TTFL oscillation, which saw a suppression of Per2 signal of ∼15% over 3 h (initial 13.2 ± 0.9%, *n* = 7) (*SI Appendix*, Fig. S1*C*) compared with the 30% decline at initiation. The differential kinetics were not sustained, however, and the time required for Per2::Luc bioluminescence to decline by 50% was not significantly different between initiation and first peak: initiation 5.5 ± 0.4 h vs. first peak 6.4 ± 0.2 h (*n* = 7), *t*(6) = 2.17, *P* = 0.07, two-tailed paired *t* test. The rapid initial decline in Per2-driven bioluminescence was likely caused by newly translated Cry1 flooding SCN neurons which would have been primed with derepressed, high levels of Cry1 mRNA ready for translation on the provision of AlkK.

Beyond its ability to initiate neuronal function, to be useful as a tool, the translational switch should be rapidly reversible. To test this, SCNs were transferred through a series of washes in culture medium lacking AlkK. Within 36 h (an artifactual immediate peak of bioluminescence caused by the wash precludes a more accurate estimation), the bioluminescent oscillations became arrhythmic [washout (w/o) in Fig. 1 *D* and *E*]. The robustness and coherence of the rhythms declined (Fig. 1 *C* and *G*), and FFT-assigned nominal periods were significantly shorter than those observed with AlkK: period, +AlkK = 28.5 ± 0.4 h vs. washout = 20.8 ± 1.6 h, *t*(8) = 4.4, *P* < 0.01; amplitude, +AlkK = 181.2 ± 59.9 vs. washout = 65.6 ± 27.6, *t*(8) = 2.6, *P* < 0.05; RAE, +AlkK = 0.06 ± 0.02 vs. washout = 0.19 ± 0.03, *t*(8) = 5.1, *P* < 0.01; GOF, +AlkK = 0.46 ± 0.07 vs. washout = 0.76 ± 0.04, *t*(8) = 5.4, *P* < 0.001; all paired *t* tests (*n* = 9). These effects indicate that as the expression of Cry1 declined its repressor activity decreased, and thereby the TTFL was disabled. Therefore the translational switch was rapidly reversible. Moreover, on repeated treatment with AlkK, a second series of clear circadian oscillations was rapidly initiated (Fig. 1 *C* and *D*), demonstrating the reproducibility of the translational switch in a tissue and the Cry1-dependent lability of ensemble SCN molecular timekeeping.

### Cellular Effects of Cry1 Translational Switching in the SCN.

**Fig. 2.**
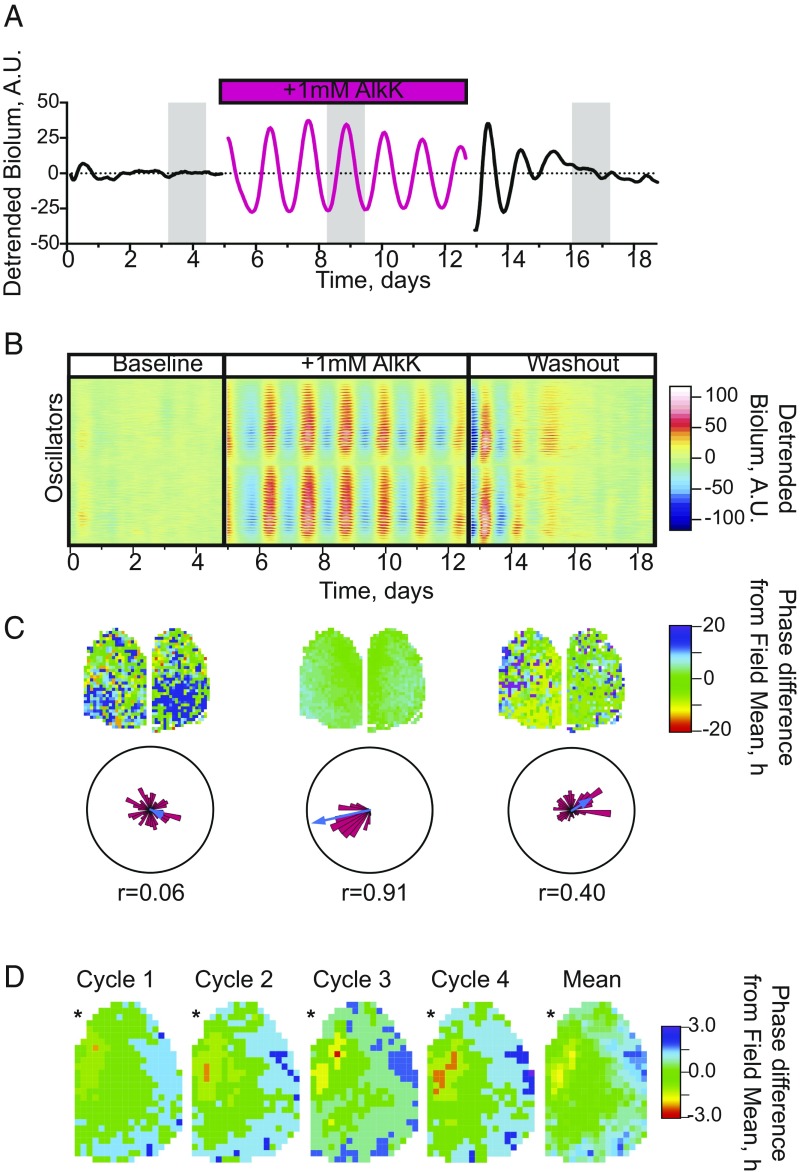
Reversible control of cell-level molecular timekeeping by translational switching of Cry1 expression in Cry-null SCN slices. (*A*) Representative normalized and detrended plot of ensemble bioluminescence, recorded by a CCD camera, from an AAV-transduced Cry1,2-null SCN slice (slice C) during baseline, treatment with 1 mM AlkK (magenta), and subsequent washout. (*B*) Raster plot of individual cellular rhythms recorded by a CCD camera from the trace shown in *A* before, during, and after treatment with 1 mM AlkK. (*C*) The corresponding mean phase-distribution maps and Rose plots for each phase of treatment, with the clustered phases of the individual cells shown in pink histograms and the vector length depicted by the blue arrow. The number below each plot depicts the Rayleigh coefficient and therefore the degree of synchrony between cells. (*D*) Phase plots of cellular oscillations across the SCN slice in *A* and *B* over the first four cycles of treatment with AlkK. Note the limited phase-dispersal of rhythmic cells in cycle 1 and the progressive dispersal over subsequent cycles as the circuit spontaneously organizes its spatiotemporal structure. The asterisk denotes the position of the third ventricle.

To examine the effects of translational switching of Cry1 expression at the level of individual SCN cells, the Per2-driven bioluminescence of AAV-transduced Cry1,2-null SCN slices was imaged by a CCD camera (Fig. 2, slice C and *SI Appendix*, Fig. S2*A*, slice B). In the absence of AlkK, the aggregate bioluminescence signal showed sporadic excursions of low amplitude (normalized amplitude 0.05 ± 0.01) and lacked any clear circadian pattern (Fig. 2*A*, slice C). This was reflected by the individual cells, which showed poorly defined circadian oscillations that were weakly coordinated across the SCN circuit (Fig. 2*B* and *SI Appendix*, Fig. S2*B*, slice B). In the absence of AlkK, FFT analysis assigned nominal periods that were distributed widely across the considered range of 15–34 h (24.6 ± 0.5 h, mean ± SEM, from four independent slices) (*SI Appendix*, Fig. S2*B* shows slice B). Indeed, in 38.61 ± 9.97% of cells, a combination of a Lomb–Scargle periodogram and FFT analysis failed to detect any statistically verifiable rhythm within the considered range. The nominal rhythms that were detected had a high RAE (0.38 ± 0.01, *n* = 4) and GOF (0.50 ± 0.01, *n* = 4) (*SI Appendix*, Fig. S2*B*), indicating poorly defined circadian properties (22). At the circuit level, widely divergent phases were evident before AlkK addition, both in phase-maps and by Rayleigh analysis (*R* = 0.39 ± 0.11, mean ± SEM) (Fig. 2*C* and *SI Appendix*, Fig. S3). This phase dispersal is characteristic of the weak cellular oscillations of a Cry1,2-null SCN.

The addition of 1 mM AlkK rapidly (by one circadian cycle) initiated strong ensemble oscillations of Per2-driven bioluminescence (Fig. 2*A* and *SI Appendix*, Fig. S2*A*). This was caused by the initiation of strong cellular oscillations (Fig. 2*B*) that exhibited FFT-determined periods converging at 29.53 ± 0.61 h, which is definitive for a Cry1-dependent TTFL (*n* = 4 slices; mean number of cells per slice = 924; range, 703–1,124 cells per slice) (*SI Appendix*, Fig. S2 *B* and *C*). The error and GOF were reduced, and the amplitude was enhanced (RAE: 0.08 ± 0.01, mean ± SEM; GOF: 0.26 ± 0.01, mean ± SEM; normalized amplitude: 0.36 ± 0.03, mean ± SEM; *n* = 4), and the cellular oscillations across the SCN were tightly synchronized both by phase-maps and by Rayleigh analysis (*R* = 0.96 ± 0.02, mean ± SEM; *n* = 4) (Fig. 2 *B* and *C* and *SI Appendix*, Fig. S3). By the fourth cycle with AlkK, the circuit had resolved into stable spatiotemporal clusters (Fig. 2*D*) comparable to the dorso-ventral spatiotemporal distribution observed in WT SCN [phase range (mean ± SEM): WT, 6.19 ± 1.13 h, *n* = 5; Cry1,2-null with AlkK: 6.25 ± 0.92 h, *n* = 4; cycle 4 WT vs. AlkK: *t*(7) = 0.04; *P* = 0.96, unpaired *t* test] (Fig. 2*D* and *SI Appendix*, Fig. S3).

Switching off translation by withdrawal of AlkK was followed by the rapid (within 36 h) loss of ensemble and cellular rhythms. FFT failed to detect rhythms in 8.6 ± 4.1% of cells after AlkK withdrawal, and the remainder showed low-amplitude rhythms with weakly defined periods distributed across the considered range (*SI Appendix*, Fig. S2*B*, slice B) (RAE: 0.41 ± 0.03, mean ± SEM; GOF: 0.73 ± 0.04, mean ± SEM; normalized amplitude: 0.09 ± 0.02, mean ± SEM; *n* = 4). This was accompanied by desynchrony across the SCN circuit evidenced by a dispersed phase-map and a fall in the Rayleigh coefficient (0.47 ± 0.18, mean ± SEM; *n* = 4) (Fig. 2 *B* and *C* and *SI Appendix*, Fig. S3). Thus, within-circuit mechanisms imposed a phase order among the individual Cry1-initiated cellular oscillators across the slice, and this order was lost on washout (Fig. 2*C* and *SI Appendix*, Fig. S3).

The reversible phase changes driven by Cry1 expression indicated that restoration of SCN cell-autonomous and ensemble rhythms involved interneuronal signaling. To test this directly, AAV-transduced slices were treated with vehicle or TTX (1 μM) to block action potential signaling, either before or after addition of AlkK (*SI Appendix*, Fig. S4). In SCN showing strong circadian bioluminescence rhythms in the presence of AlkK, the addition of vehicle had no effect on rhythm coherence, whereas TTX immediately damped the oscillation (*SI Appendix*, Fig. S4 *A* and *B*). Pretreatment with vehicle did not prevent sustained initiation of circadian rhythms by the addition of AlkK, but pretreatment with TTX (*SI Appendix*, Fig. S4 *C* and *D*) did prevent sustained high-amplitude oscillations. Importantly, in both groups there was an acute suppression followed by a TTFL-driven peak in Per2::Luc bioluminescence ∼34 h after AlkK addition (34.3 ± 0.1 h, *n* = 3 and 33.6 ± 0.8 h, *n* = 4, respectively), but subsequent oscillations were damped in the presence of TTX (*SI Appendix*, Fig. S4*E*). These immediate responses indicate that acute expression of Cry1 initiated the cell-autonomous TTFL but the absence of intercellular signaling prevented circuit-level propagation.

Overall, these results show that translational switching can be used to exert reversible and repeatable control of circadian molecular oscillations in the Cry1,2-null SCN at the level of individual cells and across the entire circuit. Thus, the neuronal and circuit-level mechanisms essential for molecular timekeeping are preestablished in the SCN despite the absence of Cry proteins during development. Expression of Cry protein, in this case Cry1, in SCN neurons is sufficient for the acute and reversible activation of the SCN TTFL and circuit-level timing, with its effects being evident after one circadian cycle.

### Translational Control of Cry1 Expression in SCN in Vivo.

**Fig. 3.**
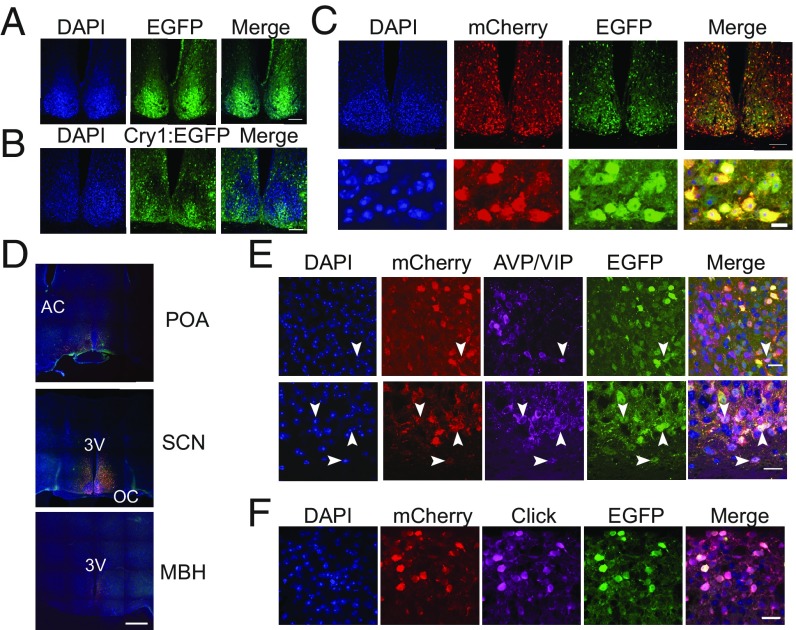
Expression of Cry1::EGFP in mouse brain by in vivo application of translational switching. (*A* and *B*) Representative low-power (20×) photomicrographs of a coronal SCN section from a Cry1,2-null mouse injected with AAV pCry1-EGFP (*A*) or pCry1-Cry1::EGFP (*B*). (Scale bar: 100 μm.) (*C*) Representative photomicrographs of a coronal SCN section from a Cry1,2-null mouse showing colocalization of the mCherry reporter of PylRS and the translationally controlled pCry1-Cry1(_177TAG_)::EGFP protein in the SCN at 20× magnification (*Upper Row*) and 63× magnification (*Bottom Row*). (Scale bars: 100 μm, *Upper Row*; 10 μm, *Bottom Row*.) (*D*) Representative 4 × 4 tiled photomicrographs of coronal sections showing the rostro-caudal extent of the AAV-mediated expression of pCry1-Cry1(_177TAG_)::EGFP in the hypothalamus. (Magnification: 20×.) 3V, third ventricle; AC, anterior commissure; MBH, medio-basal hypothalamus; OC, optic chiasm; POA, preoptic area. (Scale bar: 500 μm.) (*E*) Representative photomicrographs show the colocalization of AAV pCry1-Cry1(_177TAG_)::EGFP with the mCherry reporter of PylRS and either AVP (*Upper Row*) or VIP (*Lower Row*) immunoreactivity. (Magnification: 63×.) (Scale bars: 20 μm.) (*F*) Representative photomicrographs show the colocalizations of pCry1-Cry1(_177TAG_)::EGFP with the bio-orthogonal click label AzAF647, confirming the incorporation of AlkK into translationally switched pCry1-Cry1(_177TAG_)::EGFP. (Magnification: 63×.) (Scale bar: 20 μm.)

The slice studies validated the reagents for translational switching of Cry1 and revealed biologically appropriate oscillatory molecular rhythms in the SCN neurons and circuit. We then sought to apply it to control mouse behavior in vivo. Cry1,2-null mice received one of three AAV treatments: a control vector expressing EGFP under the minimal *Cry1* promoter (AAV pCry1-EGFP); a vector encoding a nonconditional (i.e., amber-free) construct (AAV pCry1-Cry1::EGFP) (19); or the two AAVs required for translational switching (Fig. 1*A*). Effective expression of Cry1 was determined by post hoc histological analysis of brain sections collected from mice at the end of the light phase [when endogenous Cry1 expression is high in WT mice (23)] and, in the case of the translational switch, from animals that received AlkK (30 mg/mL) in their drinking water. In mice injected with the control AAV, there was broad expression of free EGFP signal across the SCN (Fig. 3*A*). The SCN of mice injected with the nonconditional AAV (pCry1-Cry1::EGFP) exhibited nuclear-localized EGFP signal consistent with Cry1 expression (Fig. 3*B*). Comparable expression of EGFP was also evident in the SCN of Cry1,2-null mice injected with AAV encoding the conditional construct and provided with AlkK (Fig. 3*C*). Furthermore, the EGFP signal was in direct register with the neuronally localized mCherry report for the orthogonal *Pyl*T/*Pyl*S pair. The extent of pCry1-Cry1(_177TAG_)::EGFP expression was limited to the region of the SCN and surrounding hypothalamic tissue (preoptic area and medio-basal hypothalamus) (Fig. 3*D* and *SI Appendix*, Fig. S5), with 24.3 ± 2.0% (mean ± SEM) of SCN cells targeted (*n* = 15 mice). Dual immunohistochemistry performed on a subset of mice (*n* = 4) demonstrated the expression of conditional pCry1-Cry1(_177TAG_)::EGFP in both vasoactive intestinal polypeptide (VIP)-positive and arginine-vasopressin (AVP)-positive SCN neurons (68 ± 6% of VIP^+^ cells and 12 ± 3% of AVP^+^ cells were targeted) (Fig. 3*E*). A significant advantage of genetic code expansion is that the incorporated ncAA can carry specific functionality; in the case of AlkK this allowed bio-orthogonal labeling with an azido fluorophore derivative (AzAF647). Correspondingly, the pCry1-Cry1(_177TAG_)::EGFP signal was tightly in register with AzAF647 fluorescence (“click”) (Fig. 3*F*), further confirming that the translational switch worked effectively in vivo by the incorporation of AlkK into pCry1-Cry1(_177TAG_)::EGFP.

### Control of Circadian Behavior in Arrhythmic Mice by Expression of Cry1 in the SCN in Vivo.

**Fig. 4.**
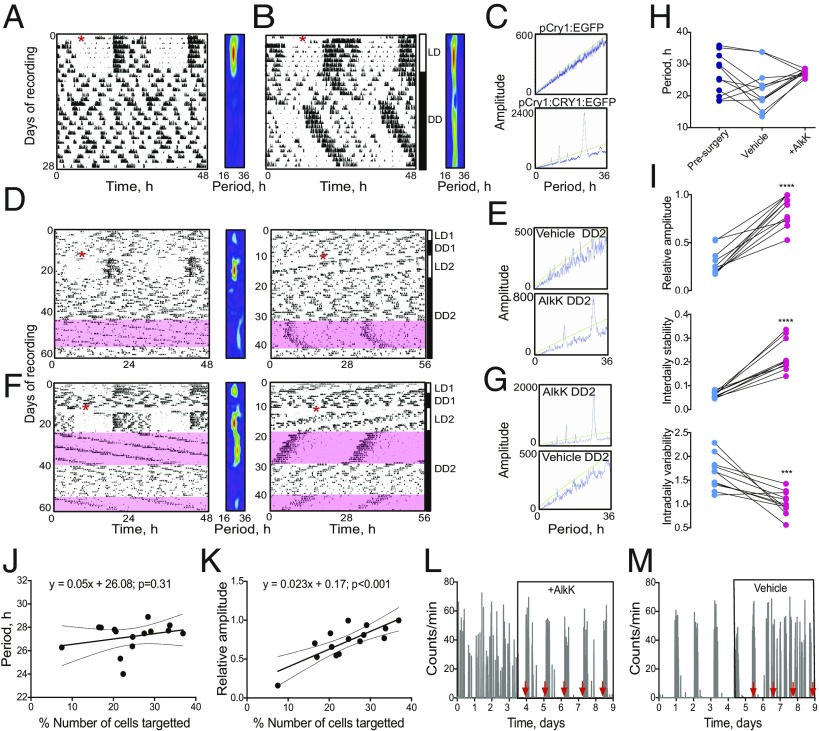
Translational switching of Cry1 expression in the SCN controls behavioral circadian rhythms in Cry-null mice. (*A* and *B*) Representative double-plotted wheel-running traces and accompanying wavelet analyses from Cry1,2-null mice injected (red asterisks) with control AAV pCry1-EGFP (*A*) or AAV pCry1-Cry1::EGFP (*B*) and transferred to DD after 7 d. (*C*) Periodogram analysis of the DD activity traces shown in *A* and *B* reveal the period-appropriate initiation of rhythmicity in the mouse injected with AAV pCry1-Cry1::EGFP. (*D*) Representative double-plotted actograms and wavelet analysis of arrhythmic Cry1,2-null mouse injected with AAV pCry1-Cry1(_177TAG_)::EGFP (red asterisk) and transferred to DD after 14 d. Mouse was provided with vehicle drinking water before the switch to 30 mg/mL AlkK (pink shaded area) and then were switched back to vehicle. The data are plotted on a 24-h time base (*Left*) and a 28-h time base (*Right*) for ease of inspection. (*E*) Periodogram analyses for the DD activity traces shown in *D* during treatment with vehicle or AlkK. (*F*) Representative actogram as in *D*, but AlkK (pink-shaded areas) was provided before and after vehicle. Data are plotted on a 24-h time scale (*Left*) and a 28-h time scale (*Right*). Corresponding periodogram analyses are shown in *G*. (*H*) Estimated circadian periods of activity rhythms of Cry1,2-null mice injected with AAV pCry1-Cry1(_177TAG_)::EGFP before surgery (navy) and then after surgery during treatment with either vehicle (Veh, light blue) or AlkK (magenta). *F*(2.20) = 3.6, *P* < 0.05, one-way repeated-measures ANOVA). (*I*) Nonparametric measures (relative amplitude, interdaily stability, and intradaily variability) of circadian behavioral rhythms of Cry1,2-null mice injected with AAV pCry1-Cry1(_177TAG_)::EGFP and then treated with either vehicle (blue) or AlkK (magenta) under DD. ***P < 0.005; ****P < 0.001; paired *t* test. (*J*) Linear regression analysis of the percentage of SCN cells expressing the AAV pCry1-Cry1(_177TAG_)::EGFP against the circadian period observed during treatment with AlkK. (*K*) As in *J*, but the relative amplitude of the initiated locomotor activity rhythm during treatment with AlkK is plotted. (*L*) Representative histogram plot of the activity of Cry1,2-null mouse injected with AAVs and initially on vehicle and then transferred to AlkK. The red arrowheads indicate clear activity onset on days 3–5, and their back-extrapolation for days 1 and 2. (*M*) As in *L*, except the transfer was from AlkK to vehicle, and activity onsets are extrapolated forward from the AlkK phase.

Local injection of nonconditional AAV pCry1-Cry1::EGFP into the SCN can lengthen the period of wheel-running behavior of short-period Cry1-null, Cry2-competent mice (19). To test whether nonconditional expression of Cry1::EGFP in the SCN is sufficient to initiate circadian behavior de novo in an otherwise arrhythmic animal, Cry1,2-null mice were injected with control AAV pCry1-EGFP or with AAV pCry-Cry1::EGFP and 10 d later were transferred to continuous dim red light (DD) to test for circadian competence. Wheel-running behavior was also recorded in WT and Cry2-null mice for comparison (*SI Appendix*, Fig. S6 *A* and *B*). Under DD, the control AAV mice exhibited no significant circadian patterning of behavior (Fig. 4*A*), although periodogram analysis was nevertheless able to assign nominal noncircadian periods (29.6 ± 1.8 h, *n* = 6) (*SI Appendix*, Fig. S6*C*). In contrast, after only a few days in DD a clearly rhythmic and very stable pattern of circadian behavior emerged in Cry1,2-null mice that received AAV pCry1-Cry1::EGFP (Fig. 4*B*). The group period was 26.2 ± 0.3 h (*n* = 6), which was not significantly different from that of the control group, due to the wide variance in the latter (*n* = 6), *t*(10) = 1.8, *P* = 0.09, unpaired two-tailed *t* test vs. AAV pCry1-EGFP. However, the circadian behavior initiated and maintained by AAV pCry1-Cry1::EGFP was significantly more robust and coherent than that in the control AAV group, as reported by the nonparametric analysis (*SI Appendix*, Fig. S6 *D*–*F*) of increased relative amplitude and interdaily stability and decreased intradaily variability: relative amplitude, *t*(10) = 7.7, *P* < 0.001; intradaily variability, *t*(10) = 4.2, *P* < 0.005; interdaily stability, *t*(10) = 5.8, *P* < 0.001; all unpaired two-tailed *t* test, *n* = 6 per group. Importantly, the circadian period expressed by mice injected with AAV pCry1-Cry1::EGFP was significantly longer than that of WT controls (23.9 ± 0.06 h, *n* = 11) and was comparable to that of Cry2-null, Cry1-competent mice (25.2 ± 0.2 h, *n* = 8), *F*(3.32) = 43.5, *P* < 0.001, one-way ANOVA; AAV pCry1-Cry1::EGFP vs. WT, *P* < 0.001, post hoc Tukey’s multiple comparisons test; AAV pCry1-Cry1::EGFP vs. Cry2-null, not significant, post hoc Tukey’s multiple comparisons test (*SI Appendix*, Fig. S6*C*) (5, 21). Thus, nonconditional expression of Cry1 in the SCN can initiate circadian behavior in otherwise clockless Cry1, 2-deficient mice and can do so with a period definitive for a Cry1-driven, Cry2-null TTFL.

### Control of Circadian Behavior by Translational Switching of Cry1 Expression in Vivo.

We then tested whether translationally switched pCry1-Cry1(_177TAG_)::EGFP could be as effective as nonconditional pCry1-Cry1::EGFP in initiating circadian behavior in arrhythmic Cry1,2-null mice. Fifteen mice injected with the conditional AAVs expressed the two fluorescent proteins, mCherry and Cry1::EGFP, in the SCN when provided with AlkK (Fig. 3*B* and *SI Appendix*, Fig. S5). One other mouse had no discernible expression in the SCN and was excluded from the behavioral analysis. Two weeks after AAV surgery, mice were transferred into DD and at the same time were given AlkK (30 mg/mL) in their drinking water or were maintained on vehicle [20% (vol/vol) artificially sweetened blackcurrant juice in water, because drinking water with AlkK is less palatable to mice]. Weekly measures of fluid intake showed that the mice drank significantly (*ca*. 55%) less when on ncAA than on vehicle treatment (*SI Appendix*, Fig. S7*A*), but this did not have a significant impact on the weekly changes in body weight of the mice (*SI Appendix*, Fig. S7*B*). In some mice (*n* = 11) circadian behavior was recorded under both vehicle and AlkK treatment, allowing within-subject analysis, while in a second group (*n* = 4) behavior was recorded under AlkK alone, following presurgical confirmation of arrhythmia.

AAV-injected mice treated with vehicle did not show circadian behavior (Fig. 4 *D*–*G*), although periodogram analysis assigned nominal periods ranging between 13 and 34 h to the vehicle-treatment phase (Fig. 4*H* and *SI Appendix*, Fig. S6*C*). In contrast, when provided with ncAA, all mice exhibited robustly rhythmic wheel-running behavior (Fig. 4 *D*–*G* and *SI Appendix*, Figs. S5 and S6*B*), and once the behavior emerged, it persisted for the duration of the treatment with ncAA. On reversal of these vehicle/ncAA contingencies, those given ncAA after vehicle initiated robust circadian behavior, whereas all mice switching to vehicle showed a breakdown of rhythmicity Fig. 4 *D*–*G*), thereby demonstrating the reversibility of the translational switch in vivo. Moreover, the effectiveness of the translational switch was repeatable within the individual mice (*n* = 5) subjected to a second cycle of AlkK provision (Fig. 4*F*).

Within individual mice, the divergent circadian periods nominally assigned during treatment with vehicle were not significantly different from the presurgery values (Fig. 4*H*). In contrast, the periods of the behavioral rhythms established by supply of AlkK converged toward 27.3 ± 0.3 h (range, 25.3–28.6 h; *n* = 11), consistent with a Cry1-dependent TTFL and significantly longer than under treatment with vehicle, *t*(10) = 2.5, *P* < 0.05, paired *t* test. The period under AlkK was also significantly longer than that of WT mice (23.9 ± 0.06 h, *n* = 11), Cry2-null mice (25.2 ± 0.2 h, *n* = 8), and mice that received nonconditional Cry1-Cry1::EGFP: period, *F*(3.32) = 43.5, *P* < 0.001, one-way ANOVA; AlkK-treated vs. WT, *P* < 0.001; AlkK-treated vs. Cry2-null, *P* < 0.001; AlkK-treated vs. AAV pCry1-Cry1::EGFP, *P* < 0.05; all post hoc Tukey’s multiple comparisons test (*SI Appendix*, Fig. S6*C*). This slower cycle may reflect a higher effective concentration of Cry1 arising from administration of the AAV vectors compared with genomically encoded Cry proteins.

Within mice, the quality of the behavioral rhythms was also significantly enhanced with AlkK compared with vehicle (*n* = 11): relative amplitude, *t*(10) = 9.3, *P* < 0.001; interdaily stability, *t*(10) = 8.4, *P* < 0.001; intradaily variability, *t*(10) = 4.5, *P* < 0.005; all paired *t* test (Fig. 4*I*). Indeed the quality of the rhythms during translational switching was comparable to that of Cry1,2-null mice that received nonconditional pCry1-Cry1::EGFP and Cry2-null mice (*SI Appendix*, Fig. S6 *D*–*F*), highlighting the potency of translationally switched Cry1. However, the amplitude and stability of the behavioral rhythms did not quite match those of WT mice, which may reflect an unidentified contribution of endogenous Cry2 to these parameters.

### Quantitative Aspects of Translationally Switched Circadian Behavior.

By combining data from the 15 AAV-injected mice that received AlkK, post hoc histological analysis showed that the period of behavioral rhythms did not vary with the number of cells targeted by AAV (Fig. 4*J*). This outcome is not surprising, given that, in the absence of systematic differences in AAV expression level, all transduced cells would express the same Cry1 protein and hence the same Cry1-dependent circadian period. In contrast, the amplitude of the behavioral rhythm was positively correlated (*P* < 0.001) with the number of targeted cells (Fig. 4*K*). The rate of transduction ranged between 8% and 38% and was associated with a range in relative amplitude from 0.1 to 1.0. This suggests that a more powerful output signal arises from an SCN circuit consisting of more functionally competent, that is, Cry1-expressing, clock neurons.

In terms of the reversibility of the effects of translational switching and the lability of circadian behavior, close inspection of the actograms revealed that initiation and dissipation of circadian timekeeping during translational switching were as rapid in vivo as seen in SCN slices in vitro. Organized circadian activity bouts and accompanying intervals of inactivity in the predicted circadian day were evident within 24–48 h following the provision of ncAA in the drinking water (Fig. 4*L*). Conversely, withdrawal of AlkK and replacement with vehicle was followed by the breakthrough of activity in the predicted circadian day, again within 24–48 h (Fig. 4*M*). The behavioral transitions for the initiation (3.0 ± 0.4 d; *n* = 6) and breakdown (2.0 ± 0 d; *n* = 6) of rhythmicity, respectively, were equally rapid: *t*(5) = 2.1, *P* = 0.09, paired *t* test, two-tailed. Finally, comparison of the mean circadian activity profiles showed that Cry1,2-null mice subject to translational switching of Cry1 expression adopted a very ordered pattern of behavior (*SI Appendix*, Fig. S8*A*) that contrasted dramatically with the disorganized profile of the same mice when treated with vehicle. Furthermore, the circadian activity profile of the mice on AlkK was directly comparable with that of WT mice, albeit with a smaller peak of activity at the start of circadian night. However, it was more clearly defined than in Cry2-null mice, in which the evening peak had an even smaller magnitude (*SI Appendix*, Fig. S8*B*).

Thus, the Cry1(_177TAG_) product expressed via the translational switch was not merely permissive for oscillation: It instructed the clockwork, and thereby behavior, to adopt a specific, Cry1-definitive circadian period. Moreover, all the relevant neural properties necessary for circadian timekeeping, from the level of cells (TTFL activation) through the organization of the SCN circuit (phase divergence, synchrony, and ensemble period) to the effective imposition of circadian order for the whole animal, were in place and ready to be expressed once Cry1 protein was available in SCN neurons. In the context of circadian behavior, therefore, the efficacy of translational switching of Cry1 expression in neurons can be considered to be specific, reversible, complete, quantitative, and rapid.

## Discussion

The current study achieved two mutually dependent aims. First, it developed and validated a biologically applicable translational switch for protein expression based on GCE to incorporate a noncanonical amino acid, AlkK, into Cry1. Second, it showed that a Cry1-competent TTFL in the SCN in an otherwise clockless mouse is sufficient to control circuit-level SCN function and circadian behavior, that this effect can be mediated solely by neurons in the SCN, and that the amplitude of the behavioral rhythm is a function of the number of circadian-competent SCN neurons. Moreover, it demonstrated that all the cell-, circuit-, and animal-level mechanisms necessary for circadian function are specified during development independently of the presence of Cry proteins.

### Translational Switching as a Neurobiological Tool.

GCE-based control of transcription has previously been developed in bacteria (24). The two-component, AAV-based system we applied for translational switching in mouse brain tissue, with the generic AAV for *PylS* and *PylT* paired with a biologically relevant protein-specific target sequence delivered by a second AAV, now provides flexibility and convenience. Importantly this protein of interest is under the control of an appropriate minimal promoter. This approach provides a broad spectrum of possibilities for manipulating protein functions and interactions in the brain using encoded ncAAs (15). These manipulations can be restricted to specific locations via cell-type–specific promoters, local AAV injection, and recombinase-directed synthetase expression. In addition, the development of strategies for the incorporation of multiple and distinct ncAAs into proteins in response to distinct codons in eukaryotic cells (25–29) may allow the incorporation of novel chemical functions into proteins; these developments may also facilitate the independent, multiplexed, and spatially and temporally controlled translational switching of different proteins within a cell or organism.

### Translational Switching Using Circadian Biology as an Output.

We showed that the effects of translational switching are readily controllable and rapidly reversible at the level of individual cells and neural circuits and in whole animals. The system thereby lends itself particularly to the analysis of dynamic biological processes based on time-critical protein synthesis and degradation, as exemplified by the circadian clock. On a practical level, repeatable and reversible translational switching was achieved by convenient changes of culture medium or drinking water. The effects in vivo were remarkably rapid despite their dependence on the drinking behavior of the mice, the bioavailability of the AlkK, and the time taken for clearance of AlkK following the switch to drinking water. At the concentration used, effective biological levels of AlkK did not persist beyond 48 h of withdrawal. This is consistent with the reported noncompartmental plasma half-life following oral gavage of 250 mg/kg (i.e., 5 mg per 20-g mouse) of AlkK being 1.09 h with an estimated *per os* clearance rate of 1.17 L⋅h^−1^⋅kg (12) and oral bioavailability being 64%. Based on these parameters, the mice in the current study drank *ca*. 3 mL AlkK/d, likely during the active phase. This represents *ca*. 4 mg bioavailable AlkK ingested/h and so is consistent with the rapid extinction of coherent behavior on withdrawal of AlkK. Nevertheless, the procedure can be refined further, not least with regard to palatability. When provided with AlkK, mice drank *ca*. 55% less, but despite the reduced volume of fluid intake, they were able to maintain body weight and exhibit vigorous behavior. Moreover, the potential for titrating the dose of ncAA, and thereby the level of Cry1 expression, is also a useful feature of the system, as it allows quantitative analysis of the control of neural activity and behavior by specific proteins. This was evident in the SCN slices in which the concentration of Cry1 and therefore the circadian period were shown to be dependent on the concentration of AlkK in the culture medium.

### Translational Switching Is Not Toxic.

The effectiveness of translational readthrough of the amber stop codon in Cry1(_177TAG_) and the consequent synthesis of the full-length protein is evidenced by the nuclear localization of EGFP (30) because both the nuclear localization sequence of Cry1 and the C-terminal EGFP are downstream of the amber codon in Cry1. Moreover, Cry1(_177TAG_)::EGFP was fully effective in initiating TTFL function, and click chemical labeling revealed the explicit incorporation of AlkK, coregistered with Cry1::EGFP, into transduced SCN neurons. A priori, it could be argued that manipulating a cell to override amber stop codons would compromise general translation and thereby disrupt the proteome and reduce cell viability. Several lines of evidence argue against this. First, the neural bioluminescent activity in SCN slices and the circadian behavior initiated by Cry1(_177TAG_)::EGFP were qualitatively and quantitatively normal, as was the morphological appearance of SCN neurons, even after several weeks of treatment. These observations are consistent with previous reports that ncAAs are selectively incorporated in response to amber codons introduced into ORFs, whereas incorporation into amber codons in endogenous 3′ termination sequences is less efficient (31). Consequently, the prolonged expression of the PylRS/tRNA pair in mice provided with AlkK does not affect their general proteome (17).

### Translational Switching as a Complement to Experimental Control of Gene Expression by Transcriptional Switches.

Notwithstanding the success of transcriptional approaches, genes of interest are commonly expressed under a heterologous promoter, and nonspecific, “leaky” background expression remains a potential confounding factor (32). Transcriptional switching therefore commonly requires an extensive breeding program to generate mice of suitable multiallelic genotypes across the multiple control groups required. Translational switching has several advantages over transcriptional switches. First, the protein of interest is under the control of its own (minimal) promoter sequences rather than the constitutively expressed promoters commonly used in other conditional systems (14). In addition, Tet- and ER-inducible systems pose problems in the reported fidelity of their control over gene expression. Control over nonspecific background expression has been improved by incorporating mRNA-destabilizing elements into the 3′ UTRs of the Tet-inducible constructs and by coordinating the expression of the transactivating proteins with repressors or by mutating the ER ligand-binding domain to control promiscuous expression. Nevertheless, the possibility that these modifications have adverse downstream effects on protein expression and on the behavior being manipulated/measured cannot be excluded. Furthermore, it is important to note that tamoxifen and particularly Tet may have potential side effects, particularly with the long-term use of an antibiotic that will affect the gut microbiome, which has been shown to influence clock function (33). Since incorporation of ncAAs via stop-codon reassignment uses the endogenous translational machinery of the host tissue, it is likely more representative of intracellular biological processes. Moreover, it has the critical advantage of reversibility, enabling more elegant, repeated-measures design in individual subjects and thereby enabling the detection of any off-target effects. As well as being independent of transcriptional switching, translational switching may also be complementary to it. Translational switching by GCE also benefits from the functionality conferred by the ncAA. In the current study we employed bio-orthogonal reactions with the encoded ncAA to confirm that AlkK was incorporated into Cry1. A wide range of functionalities, including cross-linkers, fluorophores, photo-activatable amino acids, and photo-switches, may be introduced into proteins via GCE, and these may further expand the protein-dependent activities that can be controlled by translational switches (15).

### Cry1 and the Control of Circadian Behavior.

The study provides insight into the SCN clockwork. First, it showed that expression of Cry1 in the SCN is sufficient to drive the TTFL and whole-animal circadian behavior in an otherwise clockless mouse. Second, it showed that Cry1 expression solely in neurons (via hSynapsin-mediated, neuronally restricted expression of the orthogonal tRNA synthetase) is sufficient to drive TTFL function and behavior. Recent studies have implicated astrocytic clocks in setting the period of the SCN TTFL and circadian behavioral rhythms (34, 35), but here the sufficiency of neurons to initiate and sustain SCN clock function with a Cry1-specific period is revealed. This observation chimes with the recent demonstration in loss-of-function studies that the circadian competence of SCN neurons characterized by expression of the neuropeptide neuromedin-S is necessary to control circadian behavior (36). This population constitutes *ca*. 40% of SCN neurons, whereas in the present study initiating a functional TTFL in only *ca*. 25% of SCN neurons (including both VIP^+^ and AVP^+^ neurons) was sufficient to drive circadian behavior (37, 38). Further development of the vectors, for example flexed versions to make them specific to particular SCN (and other) cell populations, will provide a powerful way to explore, in a quantitative manner, the contributions of different populations to circadian (and other) behavior.

Here we demonstrate that GCE-translational switching shows how cell-autonomous and population-level effects may tune circadian behavior. Artificial, tunable control over circadian behavior has also been demonstrated using Tet-on/off transcriptional switching for the expression of transgenic Clock (10), Per2 (14), and Bmal1 (12), although in these cases the genomic manipulations were forebrain-wide, pan-neuronal, and not targeted to the region of the SCN. Therefore transcriptional switching could be combined with translational switching to manipulate two or more TTFL factors and thereby explore protein interactions in the control of circadian behavior.

By exploiting translational switching to control the expression of Cry1 in neurons of the SCN of Cry1,2-null mice, we have demonstrated reversible and conditional control of circadian timekeeping, both in vitro and in vivo. The quality of the behavior initiated and maintained by this translationally switched Cry1 was comparable to that observed in Cry1-competent WT mice and also in Cry1,2-null mice treated with nonconditional Cry1 expression. Taken together, our findings in this study show that rhythmic Cry1 abundance may be as important to the clock as rhythmic Per2 abundance as “a critical nodal point for negative feedback within the circadian clock mechanism” (39). This effective regulation of circadian timekeeping demonstrates the reversible control of mammalian behavior using translational switching, a method of potentially broad neurobiological interest.

## Materials and Methods

### Animals and Housing.

All experiments were conducted in accordance with the UK Animals (Scientific Procedures) Act of 1986, with local ethical approval (Laboratory of Molecular Biology Animal Welfare and Ethical Review Body Committee). Cry1,2-null mice were kindly provided by G. van der Horst, Erasmus University Medical Center, Rotterdam. Per2::Luciferase mice were kindly provided by J. S. Takahashi, University of Texas Southwestern Medical Center, Dallas. All lines were maintained on a C57BL/6J background. Male mice were housed individually, and their activity patterns were assessed using running wheels and passive infrared movement detectors. Mice were entrained to a 12-h:12-h light:dark (LD) schedule (lights on at 7 AM) for at least 10 d before transfer to a schedule of continuous DD to assess their endogenous free-running period. Food and water were provided ad libitum. Data were analyzed using ClockLab (Actimetrics, Inc.) running within Matlab (MathWorks). Following surgery for intracerebral injection of the AAVs, mice were individually housed in their cages with free access to running wheels and ad libitum food and water. For translational switching, blackcurrant flavor was added to the water [vehicle: 20% (vol/vol) artificially sweetened blackcurrant-flavored water]. After 2 wk in LD, mice were transferred to constant DD. As required for translational switching, AlkK (30 mg/mL) was added to the drinking solution, and in some cases (*n* = 11) solutions were switched (vehicle to AlkK or AlkK to vehicle), so each mouse was its own control. In a second cohort of AAV-injected mice (*n* = 4) the vehicle stage was omitted. Mice were weighed, the drinking solutions were changed, and the volume of liquid drunk was measured at weekly intervals. Some mice were reexposed to AlkK to show reproducibility of the treatment (*n* = 5). All mice were placed on the AlkK treatment for the last few days of the experiment.

### Organotypic Slices.

Cultures from slices of SCN brain tissue from P10 male and female pups were prepared as previously described (40). Per2::Luc bioluminescence was recorded for 7–10 d before transduction with 1 μL (∼10^12^–10^13^ transducing units) of a 1:1 mixture of AAV pSyn1mCherry::P2A::MmPylRS and AAV pCry1::CRY1_(177TAG)_::EGFP. After a further 7–10 d of recording, slices were transferred to medium containing the AlkK (0.1–10 mM). After 7–10 d the culture medium was repeatedly changed (seven times over 2 h) to wash out the ncAA, and the bioluminescence was measured until the end of the experiment. In some cases further rounds of treatment with AlkK and washout were performed. Bioluminescence emissions from the whole slice were measured by photon multiplier tubes (Hamamatsu). For single-cell analysis, bioluminescence emissions across the slice were visualized using CCD cameras as previously described (41). Bioluminescence stacks were aligned in FIJI between treatment conditions, and the left and right SCN were delineated individually through thresholding of the image to remove extra-SCN background. Custom ImageJ scripts (written in house) were used to apply a grid of superpixels (20 × 20 µm each) across the image from which spatially tagged time-series measurements of bioluminescence intensity could be extracted and analyzed further.

### Intracerebral Injections of AAVs.

Male Cry1,2-null mice were anesthetized using isoflurane (induction, 2–4%; maintenance, 1%) with body temperature thermostatically controlled using a heating pad. Briefly, under aseptic conditions, mice received bilateral stereotaxic injections of AAV pCry1-EGFP or AAV pCry1-Cry1::EGFP (0.2 μL per site; ∼1 × 10^9^ genome copies per milliliter) or 0.3 μL of a 1:1 mixture of AAV pSyn1mCherry::P2A::MmPylRS and AAV pCry1-Cry1(_177TAG_)::EGFP (∼1 × 10^9^ genome copies per milliliter) into the region of the SCN at bregma (±0.3 mm mediolateral to bregma, 5.5 mm deep to the dural surface). AAVs were produced and purified as described (17).

### Histology.

At the end of the experiments all mice were culled between Zeitgeber time (ZT)11.5–12.5 (ZT12 is the time of lights out), a time when Cry1 expression peaks in the SCN. Brains were fixed in 4% paraformaldehyde for ∼6 h, cryo-protected in 20% sucrose overnight, cut into 40-μm sections on a freezing microtome (Bright Instruments), and mounted with Vectorshield containing DAPI (Vector Labs). Immunohistochemical analysis was performed on brains from AlkK-treated mice culled at ZT12 (*n* = 4). Brains were treated as above and then were processed for VIP (rabbit polyclonal; 1:1,000; ImmunoStar 20077) and AVP (rabbit polyclonal; 1:1,000; ImmunoStar 20069) immunoreactivity. Confocal images were acquired on a Zeiss LSM 780 confocal microscope, and images were processed in ImageJ (NIH). To assess the incorporation of the ncAA into live mice, bio-orthogonal (click) labeling was performed as previously described with azide-sulfo-Cy5 fluorophore via a Cu(I)-catalyzed cycloaddition (12, 19).

### Statistical Analysis.

Bioluminescence recordings were analyzed using BioDare software for rhythmicity and period determination (provided by A. Millar, University of Edinburgh, Edinburgh) (42). Phase was determined through peak identification within a defined time-window projected from the beginning of a treatment interval as indicated. For the slice data, at least 5 d were analyzed for each treatment. For in vivo data, the period, amplitude, and RAE data as well as the nonparametric measures using ClockLab were determined over 14 d of treatment with either the vehicle or AlkK. One- or two-way ANOVA with post hoc Tukey’s or Sidak’s multiple comparisons tests or two-tailed paired or unpaired *t* tests were used to compare differences between groups and within slices and mice.

## Supplementary Material

Supplementary File
