# Supplementary material for: Translational switching of Cry1 protein expression confers reversible control of circadian behavior in arrhythmic Cry-deficient mice
Source: Proc Natl Acad Sci U S A. 2018 Nov 28;115(52):E12388–97. doi: 10.1073/pnas.1811438115 (PMC6310849; doi:10.1073/pnas.1811438115)
Supplement: Supplementary File [file pnas.1811438115.sapp.pdf]

PNAS
www.pnas.org

Supplementary Information for

Translational switching of Cry1 protein expression confers reversible control of circadian behaviour in arrhythmic Cry-deficient mice

Elizabeth S. Maywood*, Thomas S. Elliott, Andrew P. Patton, Toke P. Krogager, Johanna E. Chesham, Russell J. Ernst, Václav Beránek, Marco Brancaccio, Jason W. Chin, Michael H. Hastings* +

Elizabeth S. Maywood, MRC Laboratory of Molecular Biology, Neurobiology Division, Francis Crick Ave., Cambridge CB2 0QH. U.K. Tel: 0044 1223 267645. Email: emaywood@mrc-lmb.cam.ac.uk

Michael H. Hastings, MRC Laboratory of Molecular Biology, Neurobiology Division, Francis Crick Ave., Cambridge CB2 0QH. U.K. Tel: 00 44 1223 267045. Email: mha@mrc-lmb.cam.ac.uk

This PDF file includes:

Figs. S1 to S8

www.pnas.org/cgi/doi/10.1073/pnas.1811438115

Supplementary Figure S1

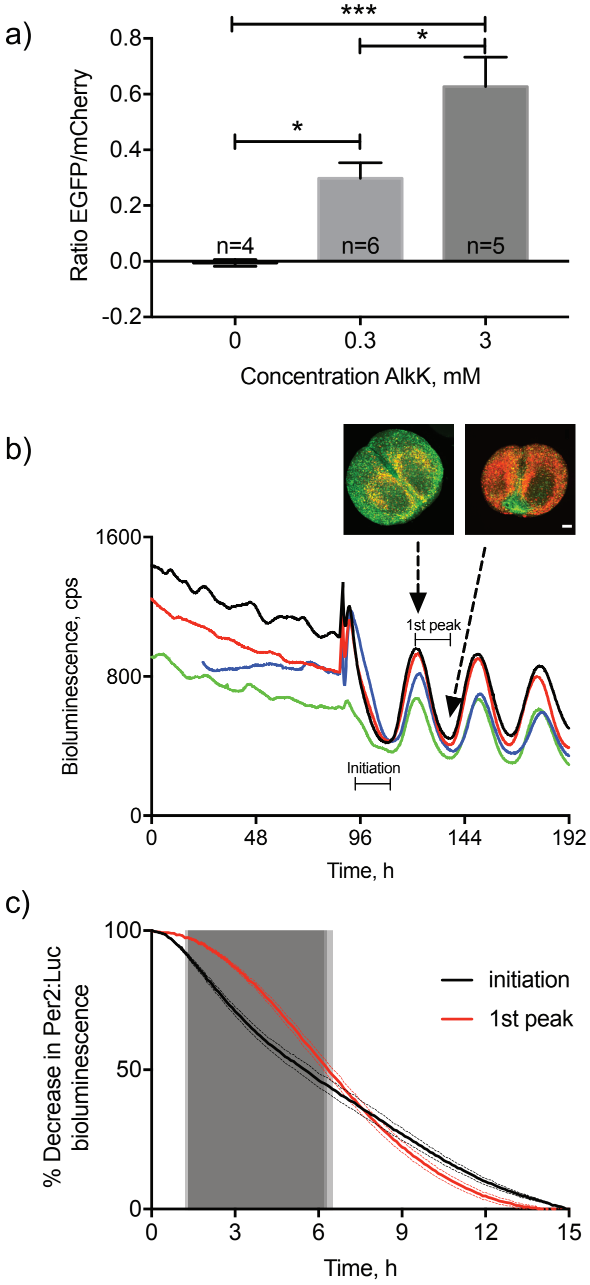

Figure S1. Control of cellular circadian rhythms by translational switching of Cry1 expression in organotypic Cry-null SCN slices.

a) Expression of Cry1::EGFP relative to mCherry shows a dose-dependent increase, representing a dose-dependent increase in Cry1 expression. Mean±SEM ratio of EGFP to mCherry (n=4-6 SCN slices per dose) following addition of vehicle (0) or AlkK (0.3, 3mM) to the medium. In each slice the average, background subtracted ratio was taken from 40 cells over 2 fields of view (63x confocal image) (1xANOVA: $F=16.95$ df 2,12 $p<0.001$; post-hoc Tukey's multiple comparisons test *$p<0.05$ 0.3mM AlkK vs vehicle; 0.3mM vs 3mM AlkK; ***$p<0.001$ 3mM AlkK vs vehicle). b) Representative traces from 4 SCN slices treated with 1mM AlkK showing the decrease in the level of Per2::Luc bioluminescence following expression of pCry1-Cry1($_{177TAG}$)::EGFP. Photomicrographs (x20; scale bar 100μm) show the colocalisation of Cry1::EGFP and mCherry (yellow/orange cells) at the peak but not at the trough of the Per2::Luc bioluminescence cycle c) Comparison between the mean (±SEM, dashed lines) decrease in bioluminescence recording from Cry1, 2-null Per2::Luc SCN slices (n=7) immediately following addition of 1mM AlkK ("initiation"; black line) and during the 1st TTFL-driven oscillation ("1st peak", red line). Grey bars represent significant differences between the "initiation" and "1st peak"; dark grey $p<0.001$; mid grey $p<0.01$; light grey $p<0.05$.

Supplementary Figure 2

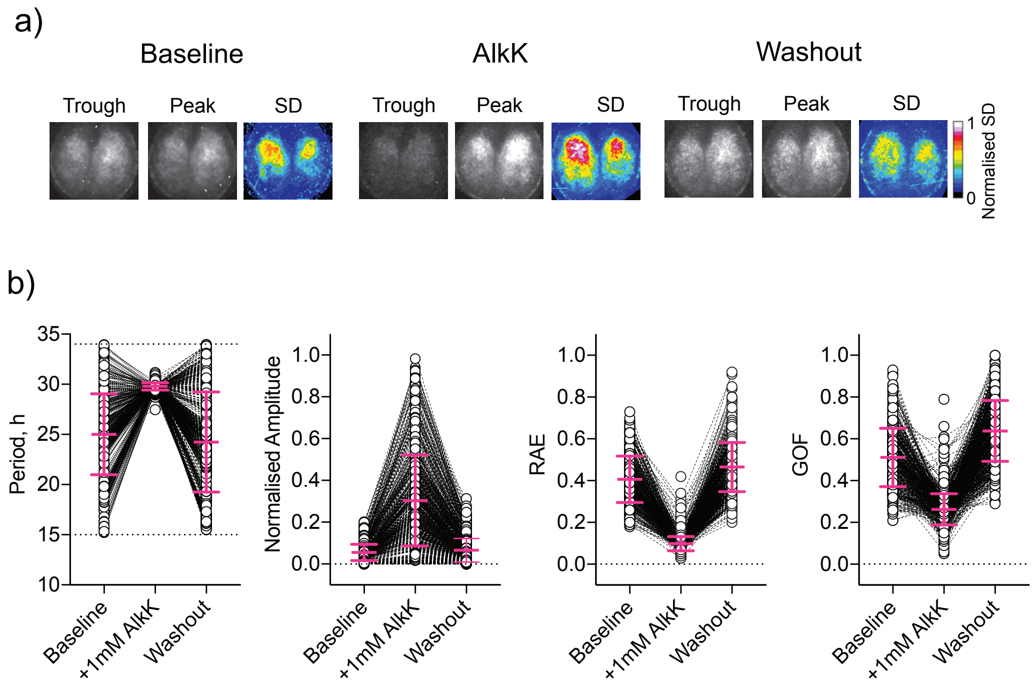

Figure S2. Control of cellular circadian rhythms by translational switching of Cry1 expression in organotypic Cry-null SCN slices.

a) Representative pixel-based images of trough and peak bioluminescence, with corresponding standard deviation of signal over SCN during pre-AlkK baseline (left), during 1mM AlkK (centre) and following wash-out of AlkK (right) (images from Slice B). b) Corresponding FFT determined period, normalised amplitude, RAE (relative amplitude error) and GOF (goodness of fit) of cellular circadian oscillations from Slice B showing the coherence of the cellular rhythms during AlkK treatment but not during baseline or washout (mean±SD shown in magenta overlayed on top of the individual values).

Supplementary Figure S3

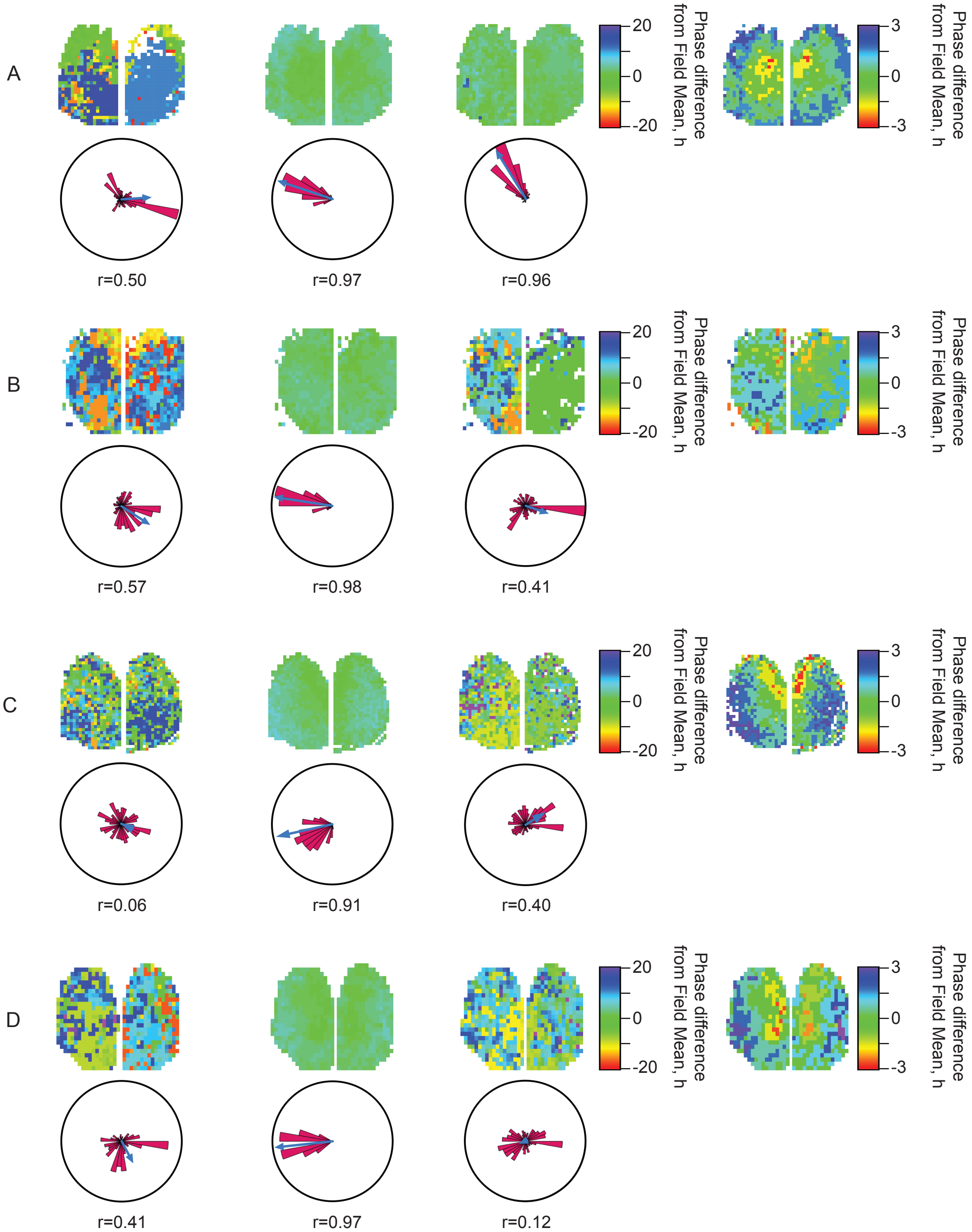

Figure S3. Control of circuit-level circadian organisation by translational switching of Cry1 expression in organotypic Cry-null SCN slices.

Phase distribution maps and associated Rayleigh plots from 4 independent Cry1, 2-null SCN slices (A-D) transduced with the AAV pCry1-Cry1($_{177TAG}$)::EGFP. Data presented during the baseline (left), 1mM AlkK treatment (centre) and washout (right). Extreme right shows phase distribution during AlkK treatment but with an expanded scale (-3 to 3h vs -20 to 20h).

Supplementary Figure S4

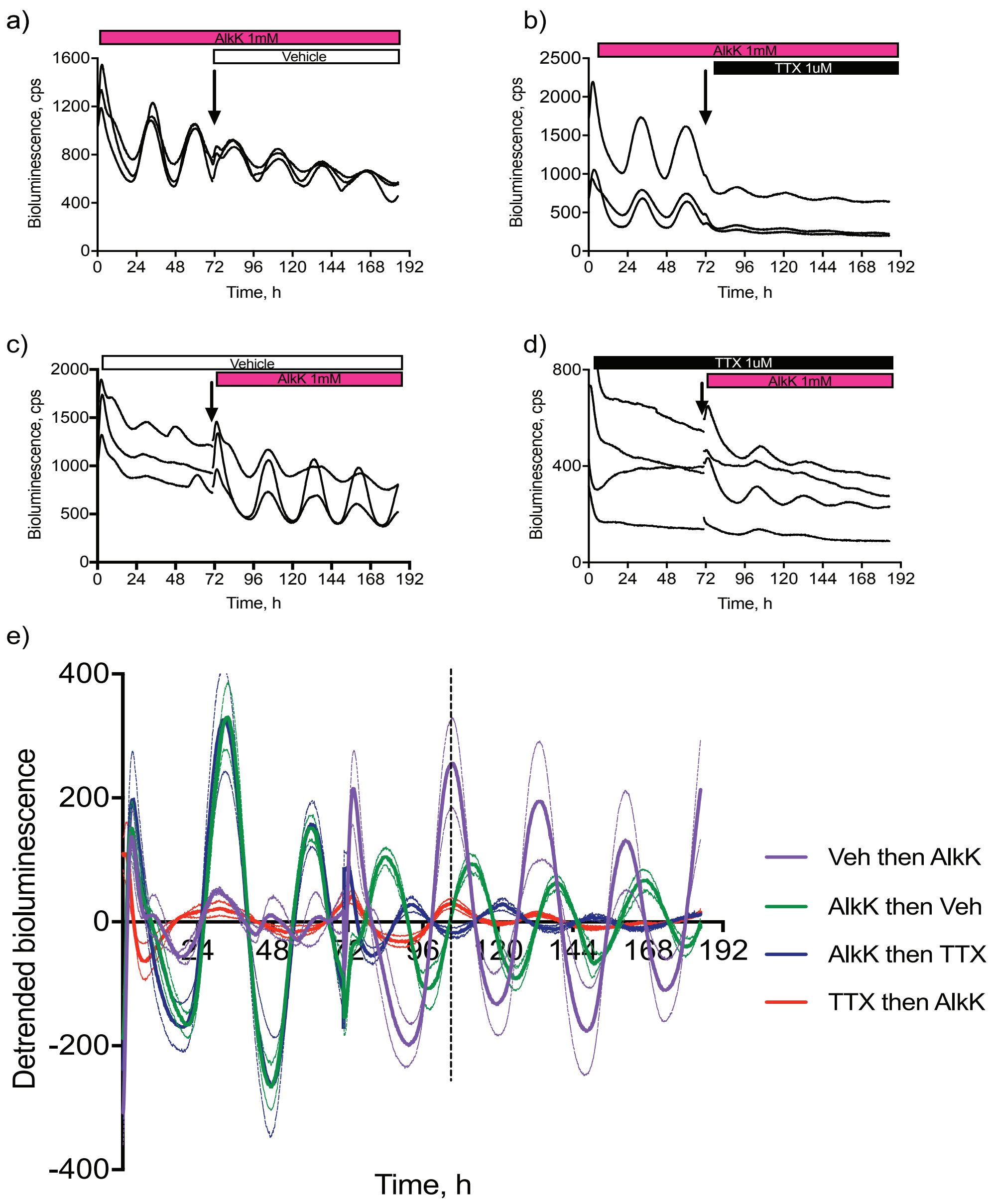

Figure S4. Control of cellular circadian rhythms by translational switching of Cry1 expression in organotypic Cry-null SCN slices is dependent on inter-cellular communication.

a) Per2::Luc bioluminescence traces from Cry1, 2-null SCN slices transduced with AAV pCry1-Cry1($_{177TAG}$)::EGFP and treated with 1mM

AlkK (magenta) followed by either vehicle or b) 1mM TTX (n=3/group). c) As in a) for slices treated with vehicle then AlkK (n=3), or d) TTX and then AlkK (n=4). e) Mean (±SEM, dashed lines) detrended bioluminescence traces from a) to d). The black dashed line aligns the 1st TTFL-driven peak following treatment with 1mM AlkK.

Supplementary Figure S5

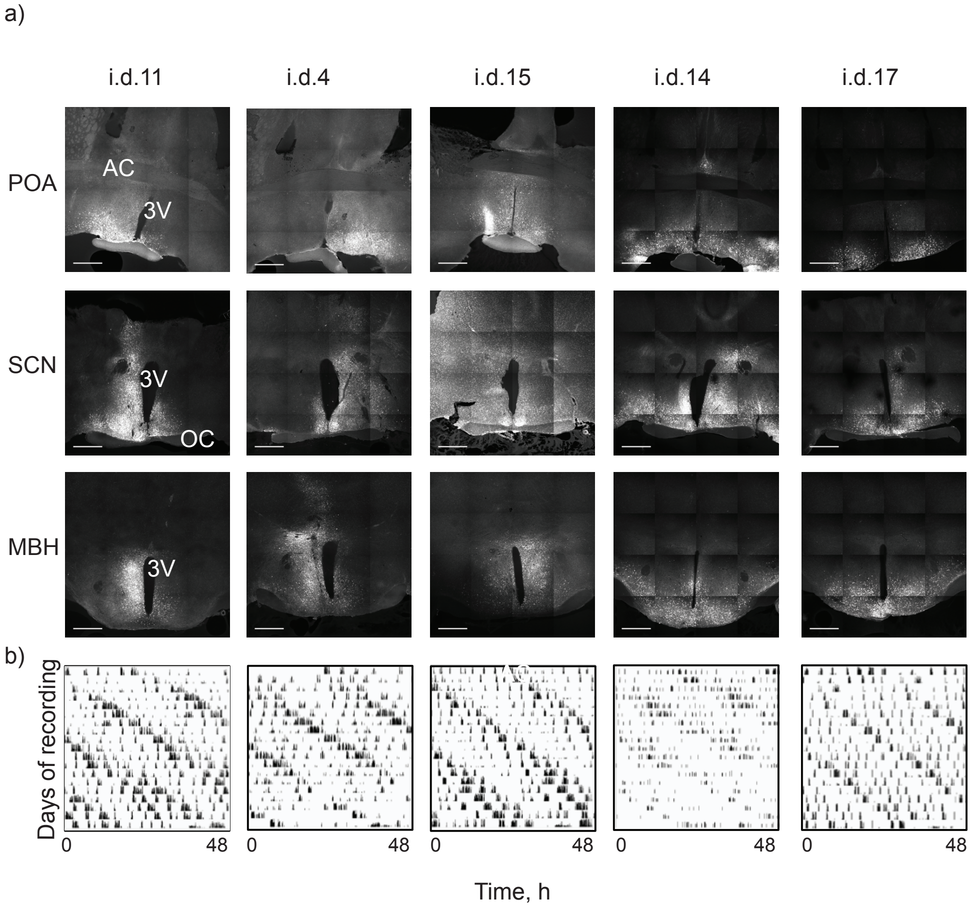

Figure S5. Translational switching of Cry1::EGFP expression in the SCN controls circadian behaviour of Cry1, 2-null mice.

a) Photomicrographs (20x magnification, 4x4 tiled) showing the rostro-caudal coronal distribution of Cry1($_{177TAG}$)::EGFP expression in the hypothalamus of 5 representative Cry1, 2-null mice (3V: 3rd ventricle; AC: anterior comminsure; OC: optic chiasm; POA: preoptic area; MBH: medio-basal hypothalamus) (Scale bar = 500μm). b) Representative double-plotted

actograms of wheel-running behaviour of the same mice as in (a) transferred to DD and provided with AlkK in drinking water. The traces are double-plotted on a 24h time base.

Supplementary Figure S6

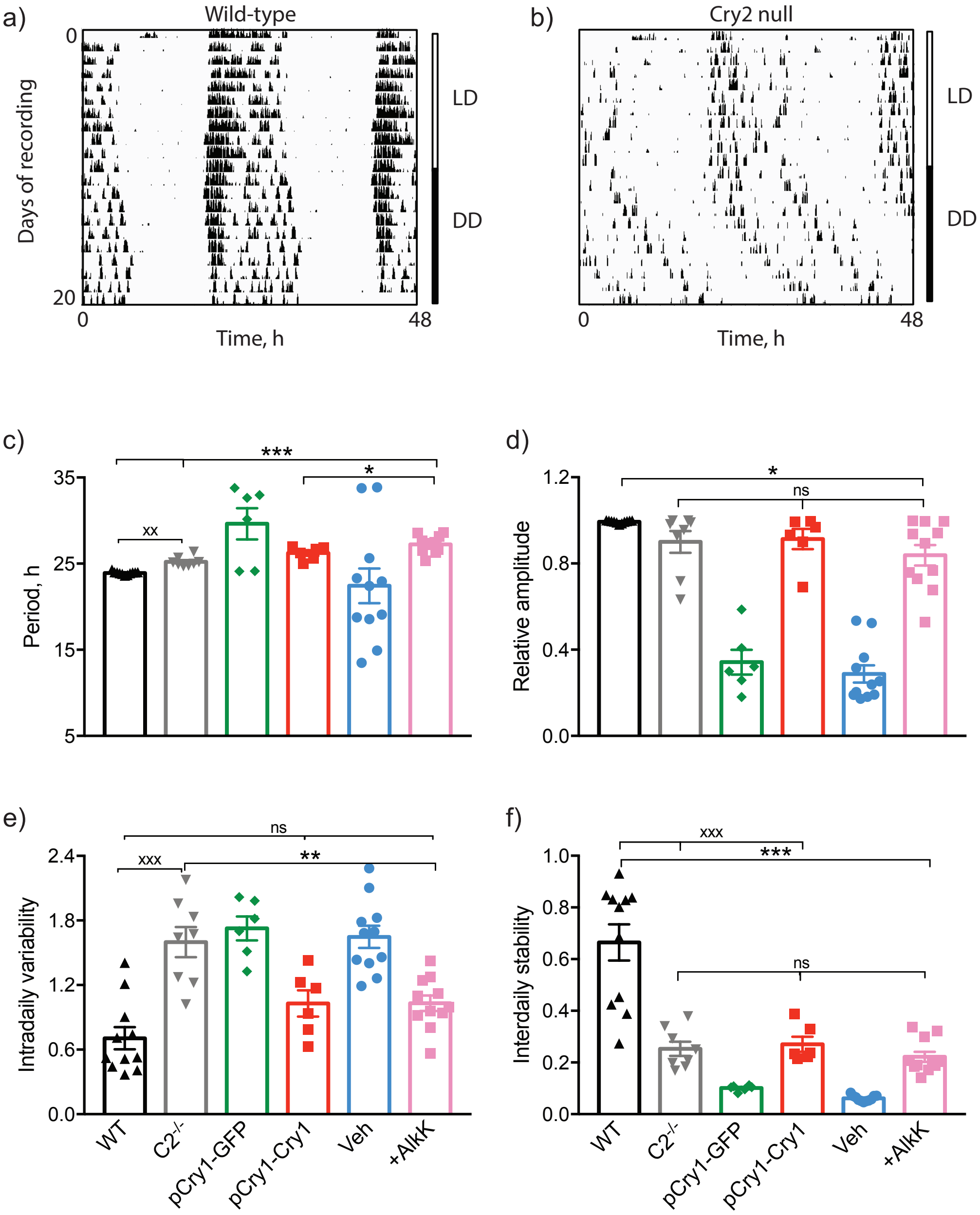

Figure S6. Quality and coherence of circadian behaviour initiated in Cry1, 2-null mice by translational switching of Cry1::EGFP expression in the SCN.

a, b) Representative double-plotted actograms of wheel-running behaviour of wild-type (a) or Cry2-null (b) mice. c- f) Group data (mean±SEM; n=6-11) for c) circadian period, d) non-parametric relative amplitude, e) intra-daily variability and f) inter-daily stability of circadian wheel-running behaviour of wild-type (black; n=11) or Cry2-null mice (grey; n=8); or Cry1, 2-null mice injected with control AAV pCry1-EGFP (green; n=6) or non-conditional pCry1-Cry1::EGFP (red; n=6); or Cry1, 2-null mice injected with AAV encoding aminoacyl-tRNA synthetase/ $tRNA_{CUA}$ pair, plus pCry1-Cry1($_{177TAG}$)::EGFP and treated with vehicle (light blue; n=11) or AlkK (magenta; n=11) for translational switching. (Period: post hoc Tukey's multiple comparison test *p<0.05 AlkK vs AAV pCry1-Cry1::EGFP; ***p<0.001 vs wild-type and Cry2-null; ^{xx}p<0.005 wild-type vs Cry2-null. Relative amplitude: 1xANOVA: F=3.2 df 3,32 p<0.05; post hoc Tukey's multiple comparison test *p<0.05 AlkK vs wild-type. Intradaily variability: 1xANOVA: F=12.1 df 3,32 p<0.0001; post hoc Tukey's multiple comparison test **p<0.005 AlkK vs Cry2-null, ^{xxx}p<0.0001 WT vs Cry2-null. Interdaily stability: 1xANOVA: F=22.4 df 3,32 p<0.0001; post hoc Tukey's multiple comparison test ***p<0.0001 AlkK vs wild-type; ^{xxx}p<0.0001 WT vs Cry2-null and AAV pCry1-Cry1::EGFP.).

Supplementary Figure S7

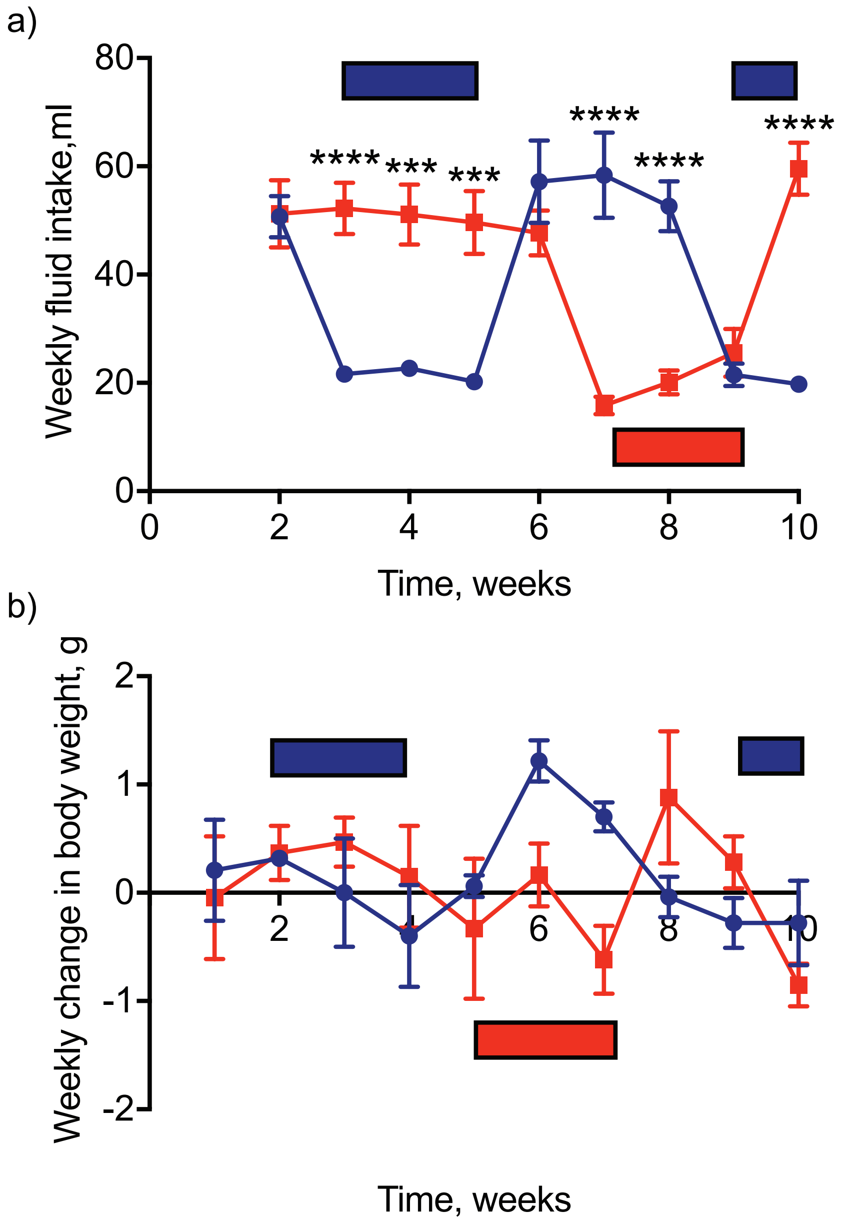

Figure S7. Effect of AlkK on drinking water consumption and maintenance of body weight.

a) Weekly fluid intake of Cry1, 2-null mice treated sequentially with AlkK, vehicle and AlkK (blue; n=5), or vehicle, AlkK and vehicle (red; n=6) in drinking water. Shaded blue and red blocks indicate phase of AlkK treatment. *** $p<0.001$ between groups (2xANOVA: Interaction F=55.0, df 8,75, $p<0.0001$; Time F=17.7 df 8,75 $p<0.0001$; Treatment F=2.0 df 1,75 $p<0.05$; post hoc Sidak's multiple comparisons test ****$p<0.0001$, ***$p<0.001$). b) Weekly change in body weight (mean±SEM), potted as in (a). (2x RM ANOVA: Interaction F=13.8, df 9,81, p=0.09; Time F=12.1 df 9,81 p=0.16; Treatment F=0.3 df 1,9 p=0.99).

Supplementary Figure S8

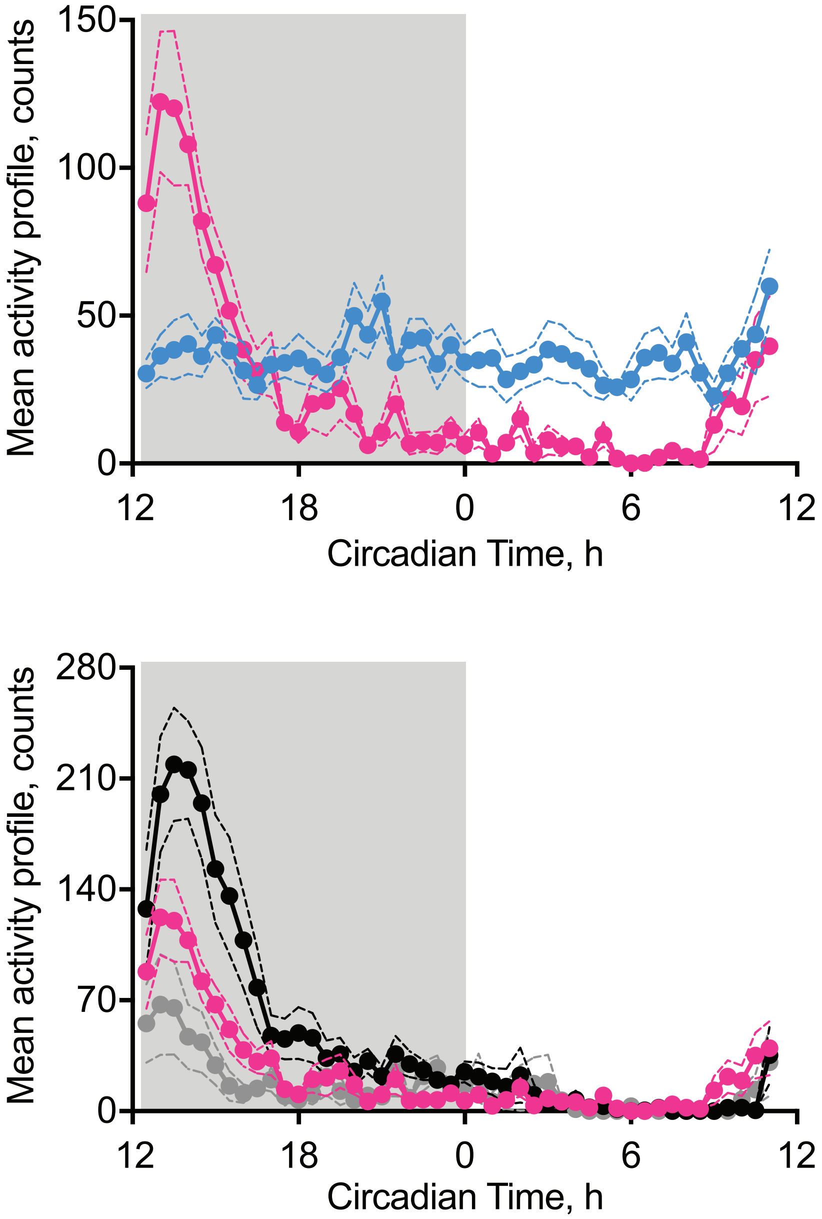

Figure S8. Comparison of circadian behavioural profiles of Cry1, 2-null mice undergoing translational switching of Cry1 expression and wild-type and Cry2-null mice.

a) Single-plotted group circadian activity profiles (mean±SEM, n =11/group) of AAV-injected Cry1, 2-null mice provided with vehicle (light blue) or AlkK (magenta) in their drinking water (Grey box denotes subjective night). b) Circadian activity profile as in (a) for AlkK-treated mice (magenta, n=11), plotted alongside wild-type (black, n=11) and Cry2-null (grey, n=8) mice. (2xANOVA RM: Interaction $F=12.5$, df 90,1035, $p<0.0001$; Time $F=32.4$, df 45,1035, $p<0.0001$; Group $F=4.5$, df 2,23, $p<0.005$.
